# Supplementary material for: Analyzing Low-Level mtDNA Heteroplasmy—Pitfalls and Challenges from Bench to Benchmarking
Source: Int J Mol Sci. 2021 Jan 19;22(2):935. doi: 10.3390/ijms22020935 (PMC7832847; doi:10.3390/ijms22020935)
Supplement: Supplementary file 1 [file ijms-22-00935-s001.pdf]

Supplementary Information

Analyzing low-level mtDNA heteroplasmy – pitfalls and challenges from bench to benchmarking

Fazzini F[1#], Fendt L[1#], Schönherr S[1], Forer L[1], Streiter G[1], Schöpf B[1], Losso JL[1], Kloss-Brandstätter A[1,2], Kronenberg F[1], Weissensteiner H[1*]

[1] *Institute of Genetic Epidemiology, Department of Genetics and Pharmacology, Medical University of Innsbruck, A-6020 Innsbruck, Austria*
[2] *Carinthia University of Applied Sciences, A-9524, Villach, Austria*

[#] *These authors contributed equally.*
** Corresponding author: hansi.weissensteiner@i-med.ac.at*

Supplementary Table S1. Averaged mixture level, standard deviation and coefficient of variation from all experiments down to 1:100. Polymerases (CLAA (Clontech), HERK and NEB). Tot DNA and PCR prod refer to the DNA extraction method, either starting from total DNA or PCR products respectively.

	M1 (1:2)						M2 (1:10)					
	Tot DNA			PCR prod			Tot DNA			PCR prod		
	CLAA	HERK	NEB	CLAA	HERK	NEB	CLAA	HERK	NEB	CLAA	HERK	NEB
**mean**	0.513	0.511	0.526	0.482	0.475	0.485	0.108	0.103	0.107	0.088	0.081	0.103
**SD**	0.032	0.036	0.032	0.036	0.030	0.035	0.011	0.013	0.012	0.010	0.011	0.012
**CV%**	6.2%	7.0%	6.1%	7.5%	6.3%	7.3%	9.8%	12.2%	11.1%	11.5%	13.5%	11.6%
	**M3 (1:50)**						**M4 (1:100)**					
	**Tot DNA**			**PCR prod**			**Tot DNA**			**PCR prod**		
	**CLAA**	**HERK**	**NEB**	**CLAA**	**HERK**	**NEB**	**CLAA**	**HERK**	**NEB**	**CLAA**	**HERK**	**NEB**
**mean**	0.022	0.021	0.023	0.018	0.017	0.020	0.012	0.011	0.013	0.012	0.010	0.011
**SD**	0.002	0.002	0.003	0.003	0.004	0.003	0.001	0.001	0.002	0.003	0.001	0.002
**CV%**	11.4%	8.4%	13.1%	18.5%	21.9%	16.7%	11.5%	12.6%	14.1%	21.7%	7.4%	15.6%

Supplementary Table S2. Low-level variants detected of the three polymerases (Clontech, Herk (Herculase) and NEB) in four different mixtures M1-M4 based on mutserve analysis (1% threshold for variant detection). See Suppl.Table 3 for details about coverage.

M1	M1-Clontech_S18	M1-Clontech_S6	M1-Herk_S1	M1-Herk_S12	M1-NEB_S21	M1-NEB_S46	M1-PCR-Clontech_S16	M1-PCR-Herk_S11	M1-PCR-NEB_S26
found out of 34	34	34	34	34	34	34	34	34	34
ignore list	4	3	3	3	4	4	3	3	4
falsPositive	4	19	0	0	22	4	22	0	8
falseNegative	0	0	0	0	0	0	0	0	0
trueNegative	16527	16513	16532	16532	16509	16527	16510	16532	16523

M2	M2-Clontech_S19	M2-Clontech_S7	M2-Herk_S13	M2-Herk_S2	M2-NEB_S22	M2-NEB_S47	M2-PCR-Clontech_S17	M2-PCR-Herk_S12	M2-PCR-NEB_S27
found out of 36	35	36	34	34	35	35	36	36	36
ignore list	4	6	4	3	7	4	7	3	6
falsPositive	5	15	1	0	16	3	18	0	12
falseNegative	1	0	2	2	1	1	0	0	0
trueNegative	16525	16512	16530	16532	16511	16527	16508	16530	16515

M3	M3-Clontech_S20	M3-Clontech_S8	M3-Herk_S14	M3-Herk_S3	M3-NEB_S23	M3-NEB_S48	M3-PCR-Clontech_S18	M3-PCR-Herk_S13	M3-PCR-NEB_S28
found out of 36	36	36	36	35	35	35	36	36	36
ignore list	5	8	3	3	6	5	5	3	6
falsPositive	3	22	2	0	8	0	30	0	14
falseNegative	0	0	0	1	1	1	0	0	0
trueNegative	16525	16503	16528	16531	16520	16529	16498	16530	16513

M4	M4-Clontech_S21	M4-Clontech_S9	M4-Herk_S15	M4-Herk_S4	M4-NEB_S24	M4-NEB_S49	M4-PCR-Clontech_S19	M4-PCR-Herk_S14	M4-PCR-NEB_S29
found out of 36	35	30	26	29	33	34	27	28	29
ignore list	7	6	3	3	8	7	5	3	8
falsPositive	4	18	1	0	13	1	20	0	9
falseNegative	1	6	10	7	3	2	9	8	7
trueNegative	16523	16515	16539	16537	16515	16527	16517	16538	16523

Supplementary Table S3. List of all the 48 samples with mean and median coverage. Performance (F1 score) shows- the median score for the 2 main analyses (impact of preprocessing at the 1% level) and low-level variant

Sample ID	Mixture	Enzyme	Mixing Source	Mean Coverage	Median Coverage	F1 Score* 1%	F1 Score# 0.4%
**H1-Clontech_S23**	1	Clontech	totDNAext	23527	25586		
**H1-Herk_S17**	1	Herculase	totDNAext	11956	12906		
**H1-NEB_S64**	1	NEB	totDNAext	4304	4607		
**M1-Clontech_S18**	1:2	Clontech	totDNAext	24910	26475	0.94	
**M1-Clontech_S6**	1:2	Clontech	totDNAext	5625	5862	0.77	
**M1-Herk_S1**	1:2	Herculase	totDNAext	6833	7033	1.00	
**M1-Herk_S12**	1:2	Herculase	totDNAext	13537	14300	1.00	
**M1-NEB_S21**	1:2	NEB	totDNAext	4206	4421	0.77	
**M1-NEB_S46**	1:2	NEB	totDNAext	6201	6568	0.94	
**M1-PCR-Clontech_S16**	1:2	Clontech	PCRprod	5159	5524	0.77	
**M1-PCR-Herk_S11**	1:2	Herculase	PCRprod	7322	7745	1.00	
**M1-PCR-NEB_S26**	1:2	NEB	PCRprod	5385	5729	0.91	
**M2-Clontech_S19**	1:10	Clontech	totDNAext	31371	32408	0.95	
**M2-Clontech_S7**	1:10	Clontech	totDNAext	6379	6828	0.82	
**M2-Herk_S13**	1:10	Herculase	totDNAext	16373	17349	0.96	
**M2-Herk_S2**	1:10	Herculase	totDNAext	5415	5630	0.97	
**M2-NEB_S22**	1:10	NEB	totDNAext	5854	6316	0.80	
**M2-NEB_S47**	1:10	NEB	totDNAext	5673	5880	0.95	
**M2-PCR-Clontech_S17**	1:10	Clontech	PCRprod	5169	5286	0.80	
**M2-PCR-Herk_S12**	1:10	Herculase	PCRprod	6061	6281	0.99	
**M2-PCR-NEB_S27**	1:10	NEB	PCRprod	6633	7112	0.86	
**M3-Clontech_S20**	1:50	Clontech	totDNAext	33925	33740	0.96	
**M3-Clontech_S8**	1:50	Clontech	totDNAext	4753	5140	0.77	
**M3-Herk_S14**	1:50	Herculase	totDNAext	15661	15951	0.99	
**M3-Herk_S3**	1:50	Herculase	totDNAext	6883	7217	0.99	
**M3-NEB_S23**	1:50	NEB	totDNAext	5910	6435	0.90	
**M3-NEB_S48**	1:50	NEB	totDNAext	7615	7935	0.99	
**M3-PCR-Clontech_S18**	1:50	Clontech	PCRprod	4125	4359	0.71	
**M3-PCR-Herk_S13**	1:50	Herculase	PCRprod	5719	5913	1.00	
**M3-PCR-NEB_S28**	1:50	NEB	PCRprod	5409	5641	0.84	

**M4-Clontech_S21**	1:100	Clontech	totDNAext	29209	30070	0.92	
**M4-Clontech_S9**	1:100	Clontech	totDNAext	5078	5295	0.71	0.06
**M4-Herk_S15**	1:100	Herculase	totDNAext	9521	10024	0.81	
**M4-Herk_S4**	1:100	Herculase	totDNAext	5434	5607	0.89	0.84
**M4-NEB_S24**	1:100	NEB	totDNAext	4473	4795	0.78	
**M4-NEB_S49**	1:100	NEB	totDNAext	6712	6965	0.96	0.07
**M4-PCR-Clontech_S19**	1:100	Clontech	PCRprod	5518	5729	0.64	0.07
**M4-PCR-Herk_S14**	1:100	Herculase	PCRprod	5830	6204	0.88	0.80
**M4-PCR-NEB_S29**	1:100	NEB	PCRprod	5154	5295	0.78	0.08
**M5-Clontech_S10**	1:200	Clontech	totDNAext	5825	6299		0.06
**M5-Herk_S5**	1:200	Herculase	totDNAext	5128	5259		0.43
**M5-NEB_S25**	1:200	NEB	totDNAext	4142	4229		0.06
**M5-PCR-Clontech_S20**	1:200	Clontech	PCRprod	3859	3188		0.06
**M5-PCR-Herk_S15**	1:200	Herculase	PCRprod	6039	6751		0.72
**M5-PCR-NEB_S30**	1:200	NEB	PCRprod	5209	5744		0.08
**U5-Clontech_S22**	1	Clontech	totDNAext	20390	21491		
**U5-Herk_S16**	1	Herculase	totDNAext	6539	6896		
**U5-NEB_S75**	1	NEB	totDNAext	2651	2751		

*** median over all preprocessing steps (fastp, MarkDuplicates, none); # median over all the variant callers**

Supplementary Table S4. Performance of the six variant callers. Sens.=Sensitivity, Spec.=Specificity and Prec. = Precision. CLAA (Clontech), HERK (Herculase) and NEB (NEB Taq).

		**M4**						**M5**					
**Performance**	**Tool**	**CLAA**	**CLAA PCR**	**NEB**	**NEB PCR**	**HERK**	**HERK PCR**	**CLAA**	**CLAA PCR**	**NEB**	**NEB PCR**	**HERK**	**HERK PCR**
Sens.	**LOFREQ**	0.94	0.86	0.97	0.92	0.89	0.94	0.64	0.50	0.61	0.64	0.47	0.39
Spec.	**LOFREQ**	0.96	0.96	0.93	0.97	0.99	0.99	0.95	0.97	0.97	0.97	0.99	0.99
Prec.	**LOFREQ**	0.05	0.04	0.03	0.06	0.14	0.16	0.03	0.03	0.05	0.04	0.08	0.07
Sens.	**GATK**	0.42	0.42	0.81	0.50	0.39	0.39	0.31	0.39	0.36	0.36	0.36	0.42
Spec.	**GATK**	0.98	0.98	0.93	0.98	1.00	1.00	0.98	0.98	0.98	0.98	1.00	1.00
Prec.	**GATK**	0.04	0.04	0.03	0.04	0.23	0.24	0.04	0.04	0.04	0.04	0.19	0.23
Sens.	**FREEBAYES**	1.00	0.97	1.00	1.00	1.00	1.00	0.83	0.92	0.89	1.00	0.81	0.44
Spec.	**FREEBAYES**	0.92	0.94	0.96	0.95	1.00	1.00	0.93	0.93	0.94	0.95	1.00	1.00
Prec.	**FREEBAYES**	0.03	0.03	0.05	0.04	0.62	0.65	0.03	0.03	0.03	0.04	0.59	0.39
Sens.	**MUTSERVE**	1.00	1.00	1.00	1.00	1.00	1.00	0.92	0.97	0.92	1.00	0.94	0.67
Spec.	**MUTSERVE**	0.89	0.90	0.92	0.90	1.00	1.00	0.90	0.89	0.89	0.91	1.00	1.00

Prec.	**MUTSERVE**	0.02	0.02	0.03	0.02	0.88	0.90	0.02	0.02	0.02	0.02	0.83	0.77
Sens.	**VARSCAN**	1.00	1.00	1.00	0.97	0.97	0.97	0.86	0.75	0.81	1.00	0.83	0.39
Spec.	**VARSCAN**	0.92	0.93	0.96	0.95	1.00	1.00	0.93	0.94	0.94	0.95	1.00	1.00
Prec.	**VARSCAN**	0.03	0.03	0.05	0.04	0.73	0.83	0.03	0.03	0.03	0.04	0.71	0.54
Sens.	**VARDICT**	0.97	0.83	1.00	0.89	0.86	0.97	0.67	0.72	0.67	0.81	0.69	0.56
Spec.	**VARDICT**	0.94	0.95	0.95	0.96	1.00	1.00	0.95	0.94	0.95	0.96	1.00	1.00
Prec.	**VARDICT**	0.03	0.04	0.04	0.04	0.94	0.97	0.03	0.03	0.03	0.04	0.89	0.83

Sens= sensitivity: Spec=specificity; Prec. = precision.

Supplementary Table S5. a) False Negatives and b) false positive variants detected. Six different variant callers applied on several mixtures of M4 (1% mixture) and M5 (0.5% mixture), for 2 different DNA extraction methods (total DNA as well starting from PCR-mixtures (PCR-prefix)), for the 3 different polymerases. CLAA (Clontech), HERK (Herculase) and NEB (NEB Taq), FN=false negatives, FP=false positives)

	M4						M5						TOTAL			MEANS		
**a)** FALSE NEGATIVES	CLAA	HERK	NEB	PCR-CLAA	PCR-HERK	PCR-NEB	CLAA	HERK	NEB	PCR-CLAA	PCR-HERK	PCR-NEB	CLAA	HERK	NEB	FN / CLAA	FN / HERK	FN / NEB
**LOFREQ**	2	2	1	5	4	3	13	22	14	18	19	13	38	47	31	9.5	11.75	7.75
**GATK**	21	22	7	21	22	18	25	21	23	22	23	23	89	88	71	22.25	22	17.75
**FREEBAYES**	0	0	0	1	0	0	6	20	4	3	7	0	10	27	4	2.5	6.75	1
**MUTSERVE**	0	0	0	0	0	0	3	12	3	1	2	0	4	14	3	1	3.5	0.75
**VARSCAN**	0	1	0	0	1	1	5	22	7	9	6	0	14	30	8	3.5	7.5	2
**VARDICT**	1	1	0	6	5	4	12	16	12	10	11	7	29	33	23	7.25	8.25	5.75
																**7.7**	**10.0**	**5.8**

	M4						M5						TOTAL			MEANS		
**b)** FALSE POSITIVES	CLAA	HERK	NEB	PCR-CLAA	PCR-HERK	PCR-NEB	CLAA	HERK	NEB	PCR-CLAA	PCR-HERK	PCR-NEB	CLAA	HERK	NEB	FP / CLAA	FP / HERK	FP / NEB
**LOFREQ**	640	173	1088	660	193	477	830	197	453	545	208	509	2675	771	2527	668.8	192.8	631.8
**GATK**	365	44	1079	397	46	410	252	49	328	351	55	349	1365	194	2166	341.3	48.5	541.5
**FREEBAYES**	1281	19	722	1062	22	852	1108	25	952	1195	20	819	4646	86	3345	1161.5	21.5	836.3
**MUTSERVE**	1867	4	1370	1712	5	1614	1691	7	1769	1847	7	1567	7117	23	6320	1779.3	5.8	1580.0
**VARSCAN**	1262	7	686	1095	13	876	1139	12	958	1032	12	867	4528	44	3387	1132.0	11.0	846.8
**VARDICT**	1070	1	829	773	2	706	826	4	821	925	3	734	3594	10	3090	898.5	2.5	772.5
																**996.9**	**47.0**	**868.1**

Supplementary Methods

Analysis of mixtures M1-M4 with mutserve (v.1.3.4)

Based on an average per base coverage of approximately ~8,900x for Q20 data, the sensitivity for detection of minor mixture components most prominently showed alignment-issues around sites 151 and 152. Since mtDNA-Server applies the per Base Alignment Quality (BAQ)[8] as alternative to realignment, these 2 SNPs mostly get filtered out by falsely interpreted as misalignment. Therefore BAQ was disabled. Further indels around homopolymeric C-stretches were filtered so that we ignored positions around homopolymeric stretches 302-309 and 311-15, around the AC repeat at position 523-524 and around 3107 due to the N on the reference sequence, resulting in issues for some variant caller. Also private low-level mutations where ignored in the analysis, if present at ~1% in the source sample. For all mixtures the threshold for heteroplasmic detection was set to 1% variant allele frequency. Here most prominently the sensitivity differed depending on the polymerase used. Single false positive mutations did not exceed levels of 1.2% in each mixture type, except for variant on position 3210 with up to 8.6% (classified as phantom mutation, as found only in NEB mixtures M1-M4, but not in NEB source samples H1 and U5). All 48 samples where processed via 3 workflows:

1. **default**: FASTQ -> BWA MEM -> SAMTOOLS -> BAM -> Picard-Tools AddOrReplaceReadGroups -> mutserve v.1.3.4
2. **fastp**: FASTQ -> FASTP -> BWA MEM -> SAMTOOLS -> BAM -> Picard-Tools AddOrReplaceReadGroups -> mutserve v.1.3.4
3. **markdup**: final BAM from 1.default -> Picard-Tools MarkDuplicates -> BAM -> mutserve v.1.3.4

The parameters for mutserve: **`java -jar –Xmx8G mutserve-1.3.4.jar analyse-local --input BAM_folder --output mutserve1.3.4_0.01.vcf --level 0.01 --noBaq --reference reference/rcrs.fasta`**

Comparative data analysis for Mixtures M4 and M5

In order to compare all variant for their low-level variant calling performance, the paired FASTQ files for all mixtures M4 and M5 were aligned/mapped to the mitochondrial reference sequence rCRS *with BWA-MEM*[10] *version 0.7.15-r1140* and the BAM files generated, sorted and indexed by *samtools v.1.10,* without pre-processing steps (i.e. no fastp and no duplication removal). Supplementary Table 2 lists all variant callers employed with URL to source code, Version, information, as well as the programming language. All variant callers where installed and run on an Linux Instance with CentOS 7.8.2003 from April 2020 on an Intel(R) Xeon(R) CPU E5-2640 v4 @ 2.40GHz with 8 Cores and 32GB RAM. Total Sample size was 870 MB with a mean and

median sample coverage (based on mutserve) of 5,855 and 5,865 over all 16569 positions in all 12 mixtures.

Supplementary Table S6. List of all variant callers employed with URL to source code / binaries, version, programming language and wall time needed for the analysis of one sample on an CentOS machine with.Intel Xeon CPU E5-2640 v4 @ 2.40GHz and 32 GB RAM.

TOOL	URL	Latest Version (release Date)	Progr. Language	Time per sample
freebayes	https://github.com/ekg/freebayes	1.3.2 (December 2019)	C++	04:37 sec
GATK4 mutect2	https://github.com/broadinstitute/gatk	4.1.8.1 (July 2020)	Java / Python	05:16 sec
LoFreq*	https://github.com/CSB5/lofreq	2.1.5 (June 2020)	C / Python	01:43 sec
mutserve	https://github.com/seppinho/mutserve	1.3.4 (May 2020)	Java	00:35 sec
VarDictJava	https://github.com/AstraZeneca-NGS/VarDictJava	1.7.0 (October 2019)	Java / Shell	02:41 sec
Varscan	https://github.com/dkoboldt/varscan	2.4.4 (July 2019)	Java	00:44 sec

- **freebayes:** freebayes is a Bayesian haplotype-based generic variant detector for NGS data, not limited to SNPs, but also able to detect insertions and deletions as well as shorter structural variants like multinucleotide polymorphisms and complex events, being very popular (over 1,500 citations on Google Scholar as of August, 2020). We used the precompiled version 1.3.1, as 1.3.2 ensures compatibility with Python 3, but otherwise very similar to 1.3.1. We run freebayes by looping over all M4 and M5 BAM files with the parameter as below, with a runtime per sample ~

```
for i in *; do
freebayes-v1.3.1 -f reference/rcrs.fasta -b $i --min-mapping-quality 30 --min-base-quality 30 --min-alternate-fraction 0.004 --min-alternate-count 5 --ploidy 2 --region rCRS:1-16569 > "$i".freebayes.vcf;
done
```

- **GATK4 Mutect2:** GATK is very popular, and with over 7,600 citations (August, 2020) the most cited of all variant callers in this validation. It comes with a large selection of useful tools for managing NGS data and even enables the creation of own pipelines. With one of the later versions, GATK4's Mutect2 has an mitochondrial DNA calling mode

included. We ran it over all BAM files M4 and M5 (total 12 samples) with the following parameters:

```
for i in *; do
gatk Mutect2 -R reference/rcrs.fasta -L rCRS --min-base-quality-
score 30 --mitochondria-mode -I $i -O "$i".gatk.vcf;
done
```

- **LoFreq:** LoFreq takes the sequence quality into consideration, when performing the ultra-sensitive variant calling. It can be used for SNP and indel calling, and quality measures into considerations, and can be used as a generic variant caller. Already the first version of LoFreq (cited over 500 times as of August 2020) was evaluated with mitochondrial genomes. Here we run the latest version LoFreq 2.1.5 over all samples:

```
for i in *; do
lofreq call -f reference/rcrs.fasta -o "$i"_lofreq.vcf -B $i --
minimum-mapping-quality 30
done
```

- **mutserve*:*** mutserve is the updated core of mtDNA-Server (cited over 70x, as of August 2020 on Google Scholar) which was designed specifically for the human mitochondrial genome in the first versions. This changed with the last versions, so that it can also be applied to different reference sequences. The detailed list of internal parameters can be found in the manuscript as well as on the mtDNA-Server help page (https://mtdna-server.uibk.ac.at/index.html#!pages/help). We ran mutserve with the parameters as follows, as it is one of the few variant caller also allowing to use it on folders containing the BAM/CRAM files (here assuming M4M5bamFolder contains all mixtures):

```
java -jar –Xmx8G mutserve-1.3.4.jar analyse-local --input
M4M5bamFolder --output mutserve1.3.4_0.004.vcf --level 0.004 --
noBaq --reference reference/rcrs.fasta --baseQ 30
```

- **VarDict** Similar to LoFreq, Vardict (cited ~280x) was designed to accurately detect variants in next-generation sequencing (NGS) data focusing on cancer genomes. It is able to call single as well as multiple nucleotide variants, indels (insertion and deletions) as well as more complex structural variants[3]. It comes with its own deduplication step, allows for detection of PCR artefacts and performs local realignments. For the validation within this

project, the JAVA version of VarDict was applied on the sorted BAM files, with the allele frequency threshold of 0.4%.

```
AF_THR=0.004
for i in *; do
bedtools bamtobed -i $i > "$i".bed;

java -jar VarDictJava/build/libs/VarDict-1.7.0.jar -G
reference/rcrs.fasta -f $AF_THR -N $i -q 30 -b $i -h -c 1 -S 2 -E
3 -g 4 -R rCRS:1-16569 "$i".bed
VarDictJava/VarDict/teststrandbias.R |
VarDictJava/VarDict/var2vcf_valid.pl -N $i -E -f $AF_THR >
"$i".vardict.vcf;
done
```

- **VarScan2** Koboldt et al published Varscan (in 2009) and Varscan2 (in 2012), cited in total almost 4,000 times, underlining its acceptance in the scientific community. It was shown that for coverage of 1,000x VarScan2 was best in discovering variants present at 1%, compared to somatic variant callers[11]. VarScan2 accepts the pileup file generated from the BAM file with SAMtools[12]. As SAMtools mpileup applies coverage filtering of 8,000x, this parameter needs to be overwritten e.g. (-d 100000) if a coverage of 100,000 should be accepted. Having a mean coverage ~6,000x we did not apply this parameter. The following parameters where used for VarScan2 (version 2.4.4 applied) over all BAM files:

```
for i in *; do
samtools mpileup -B -f reference/rcrs.fasta $i | java -jar
VarScan.v2.4.4.jar mpileup2snp --min-var-freq 0.004 --min-avg-
qual 30 --min-reads 2 5 --min-coverage 10 –output-vcf >
"$i".varscan.vcf;
done
```

As all tools emit VCF files subsequently “bcftools query” can be applied to extract the information about variant position, reference base, alternative base, coverage and allele frequency, such that the tools can be compared. All files where merged and checked manually in LibreOffice, in order to annotate sites to ignore, (around homopolymeric-C stretches 302-309 and 311-15, around the AC repeat at position 523-524 and around 3107 due to the N on the reference sequence, as reported previously). Subsequently the files were analyzed in RStudio, using the reshape2 library, and ggplot2 library for the generation of the figures.

Calculation of definitions for performance

Performance metrics were calculated according to Weissensteiner *et al.*[1]

Sensitivity =number of true positives/(number of true positives + number of false negatives)

Specificity= number of true negatives/(number of true negatives + number of false positives)

Precision= number of true positives/(number of true positives + number of false positives)

F1 Score = 2 * (Precision * Sensitivity) / (Precision + Sensitivity)

Supplementary Table S7. List of all variants defining the gold-standard. Carrier "H1" and "U5" denote the two samples. "Shared" indicates that the specific variant is present as homoplasmy in the mixture. CLAA = Clontech, Herk = Herkulase, NEB = NEB Taq. "DNA" indicates mixture from total DNA, while PCR indicates that the mixtures were performed on the PCR products. In total 24 mixtures starting from DNA and 12 from PCR products are represented, whereby the numbers indicate the amounts the variant was present. The private low-level variants down to 1% (see comments) were confirmed with HiSeq sequencing. The table additionally lists sites which were ignored for the analysis – either due to issues or too low heteroplasmic levels – not detectable in all mixtures (see comments).

Pos	Ref.	Alt.	Carrier	Gold-stand	ClAA DNA	Herk DNA	NEB DNA	CLAA PCR	Herk PCR	NEB PCR	Total Sum	H1	U5	Comment
**73**	A	G	U5	Yes	8	8	8	4	3	3	34	0	3	
**151**	C	T	U5	Yes	7	7	8	4	4	3	33	0	3	
**152**	T	C	U5	Yes	8	7	8	4	4	4	35	0	3	
**263**	A	G	Shared	Yes	8	8	8	4	4	4	36	3	3	
**477**	T	C	H1	Yes	8	8	8	4	4	4	36	3	0	
**750**	A	G	Shared	Yes	8	8	8	4	4	4	36	3	3	
**1438**	A	G	Shared	Yes	8	8	8	4	4	4	36	3	3	
**2706**	A	G	U5	Yes	8	8	8	3	4	4	35	0	3	
**3010**	G	A	H1	Yes	8	8	8	4	4	4	36	3	0	
**3197**	T	C	U5	Yes	8	8	8	4	4	4	36	0	3	
**3768**	A	G	U5	Yes	8	8	8	4	4	3	35	0	3	
**4769**	A	G	Shared	Yes	8	8	8	4	4	4	36	3	3	
**5979**	G	A	H1	Yes	8	8	8	4	4	4	36	3	0	
**7028**	C	T	U5	Yes	8	8	8	3	4	4	35	0	3	
**8860**	A	G	Shared	Yes	8	8	8	4	4	4	36	3	3	
**9145**	G	A	U5	Yes	8	8	8	4	3	4	35	0	3	
**9462**	T	C	H1	Yes	6	5	6	3	3	3	26	3	0	H1 private mutation (1.3%)
**9477**	G	A	U5	Yes	7	7	8	4	4	4	34	0	3	
**11467**	A	G	U5	Yes	8	7	7	4	3	4	33	0	3	
**11719**	G	A	U5	Yes	8	7	8	4	3	4	34	0	3	
**12308**	A	G	U5	Yes	8	6	8	3	4	4	33	0	3	
**12372**	G	A	U5	Yes	8	7	8	4	4	4	35	0	3	
**13617**	T	C	U5	Yes	8	8	8	4	4	4	36	0	3	

**14766**	C	T	U5	Yes	7	8	8	3	4	3	33	0	3	
**14793**	A	G	U5	Yes	8	8	8	3	4	4	35	0	3	
**15236**	A	G	H1	Yes	6	2	7	3	3	3	24	3	0	H1 private mutation (1.2%)
**15289**	T	C	U5	Yes	8	8	8	4	4	4	36	0	3	
**15326**	A	G	Shared	Yes	8	8	8	4	4	4	36	3	3	
**15372**	T	C	U5	Yes	2	2	2	1	1	1	9	0	3	U5 private mutation (4%)
**16129**	G	A	H1	Yes	5	3	2	3	3	3	19	3	0	H1 private mutation (1%)
**16189**	T	C	U5	Yes	8	7	8	3	3	4	33	0	3	
**16234**	C	T	U5	Yes	7	7	7	3	3	3	30	0	3	
**16256**	C	T	U5	Yes	7	6	8	3	4	3	31	0	3	
**16270**	C	T	U5	Yes	6	7	6	3	3	3	28	0	3	
**16311**	T	C	U5	Yes	8	7	8	4	4	4	35	0	3	
**16362**	T	C	U5	Yes	8	8	8	4	3	4	35	0	3	
**16526**	G	A	U5	Yes	8	8	8	4	4	4	36	0	3	
310	T	C	Shared	**Ignore**	8	8	8	4	4	4	36	3	3	Issues homo-polymeric stretch
513	G	A	Shared	**Ignore**	1	0	1	0	0	0	2	0	0	Issues 523-24 AC repeat
3065	T	C	H1	**Ignore**	1	0	2	1	0	1	5	0	0	Prob ~ 0.5% privat mut
3106	C	A		**Ignore**										Reference Issues
3106	C	G		**Ignore**										Reference Issues
3106	C	T		**Ignore**										Reference Issues
3107	N	C		**Ignore**										Reference Issues
7076	A	G	H1	**Ignore**	5	0	5	2	0	3	15	2	0	Prob 0.8-0.9% privat mut
11150	G	A	H1	**Ignore**	1	0	3	1	0	2	7	1	0	Prob 0.5% privat mut
15635	T	C	H1	**Ignore**	5	0	3	3	0	2	13	1	0	Prob 0.8-0.9% privat mut

Supplementary Figure S1. An example of an agarose-gel for 9 samples using the NEB Taq polymerases, including two negative controls and two positive controls (marked in red and green boxes, respectively).

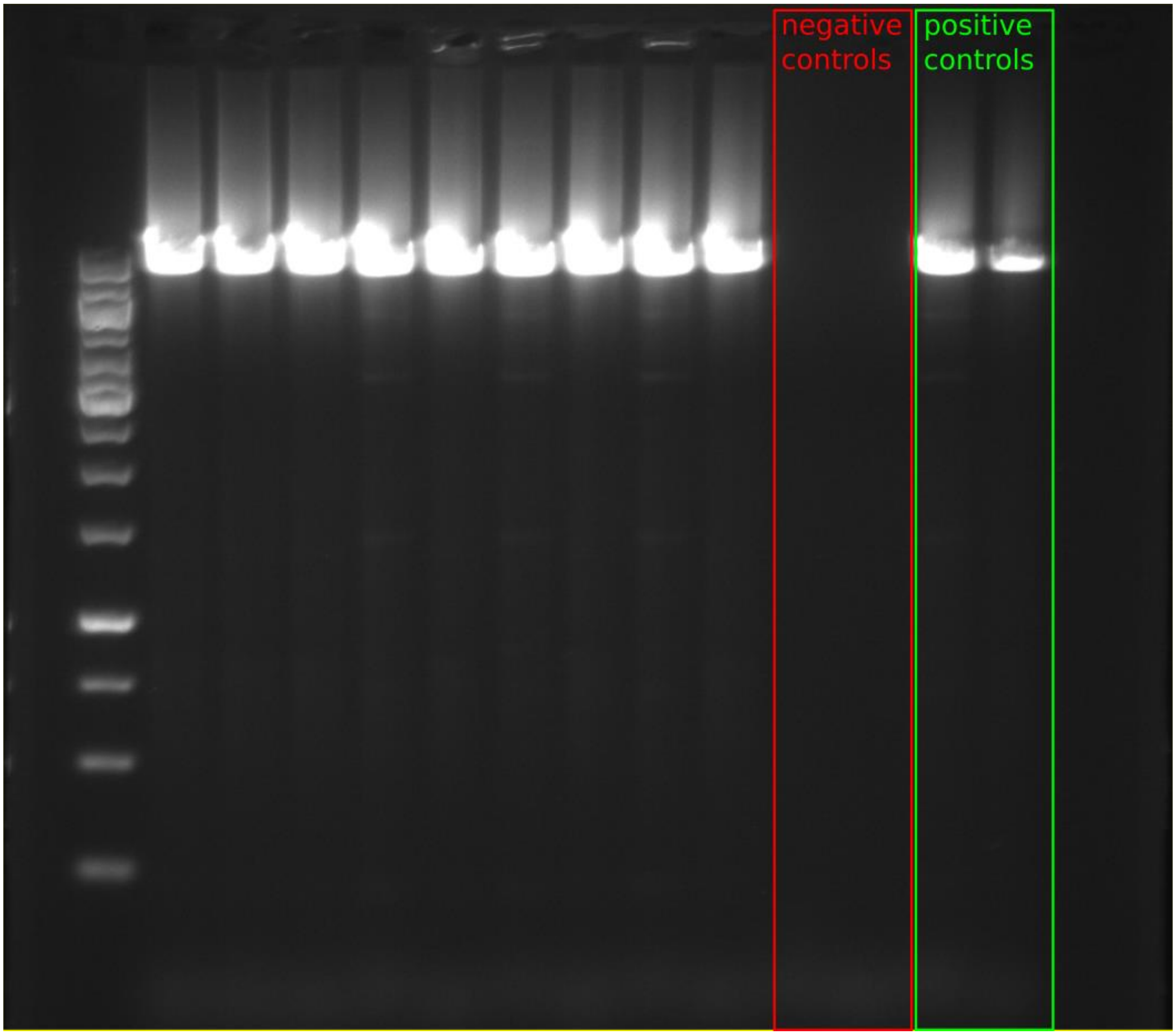

Supplementary Figure S2. Nucleotide distribution over all false positive variants. Represented are the nucleobase changes (e.g. AG indicating a change from base A-to-G) for the 1% mixtures (M4). Interestingly most changes are A to G (~34%) and T-to-C (~65%). While C-to-T base changes would be expected far more often, only few exceptions are present. rCRS expected variants are included for comparison.

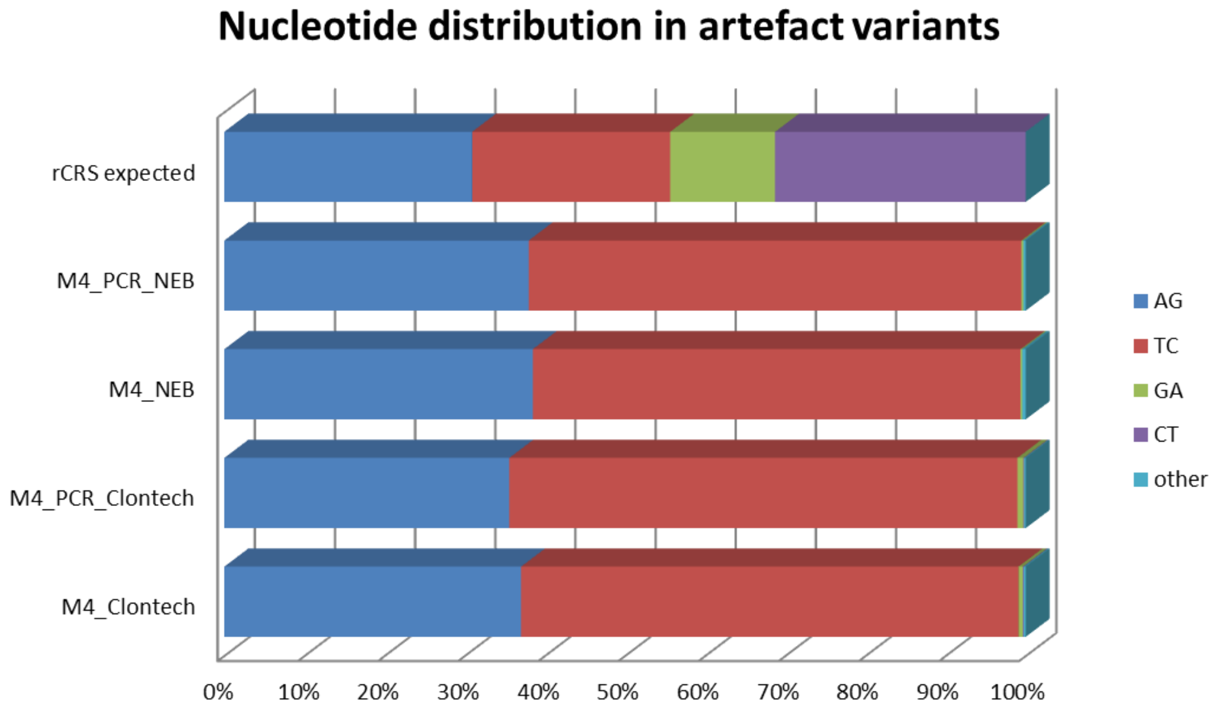

References

1. Weissensteiner, H. *et al.* mtDNA-Server: next-generation sequencing data analysis of human mitochondrial DNA in the cloud. *Nucleic Acids Res.* **44**, W64–W69 (2016).

2. Wilm, A. *et al.* LoFreq: a sequence-quality aware, ultra-sensitive variant caller for uncovering cell-population heterogeneity from high-throughput sequencing datasets. *Nucleic Acids Res.* **40**, 11189–11201 (2012).

3. Lai, Z. *et al.* VarDict: A novel and versatile variant caller for next-generation sequencing in cancer research. *Nucleic Acids Res.* **44**, (2016).

4. Koboldt, D. C. *et al.* VarScan 2 : Somatic mutation and copy number alteration discovery in cancer by exome sequencing. 568–576 (2012). doi:10.1101/gr.129684.111

5. Garrison, E. & Marth, G. Haplotype-based variant detection from short-read sequencing. (2012). Available at: http://arxiv.org/abs/1207.3907. (Accessed: 21st October 2019)

6. McKenna, A. *et al.* The genome analysis toolkit: A MapReduce framework for analyzing next-generation DNA sequencing data. *Genome Res.* **20**, 1297–1303 (2010).

7. Poplin, R. *et al.* Scaling accurate genetic variant discovery to tens of thousands of samples. *bioRxiv* 201178 (2017). doi:10.1101/201178

8. Li, H. Improving SNP discovery by base alignment quality. *Bioinformatics* **27**, 1157–8 (2011).

9. Andrews, R. M. *et al.* Reanalysis and revision of the Cambridge reference sequence for human mitochondrial DNA. *Nature Genetics* **23**, 147 (1999).

10. Li, H. Aligning sequence reads, clone sequences and assembly contigs with BWA-MEM. 3 (2013).

11. Stead, L. F., Sutton, K. M., Taylor, G. R., Quirke, P. & Rabbitts, P. Accurately identifying low-allelic fraction variants in single samples with next-generation sequencing: Applications in tumor subclone resolution. *Hum. Mutat.* **34**, 1432–1438 (2013).

12. Li, H. *et al.* The Sequence Alignment/Map format and SAMtools. *Bioinformatics* **25**, 2078–2079 (2009).
